# Development of platensimycin, platencin, and platensilin overproducers by biosynthetic pathway engineering and fermentation medium optimization

**DOI:** 10.1093/jimb/kuae003

**Published:** 2024-01-23

**Authors:** Lucas L Fluegel, Ming-Rong Deng, Ping Su, Edward Kalkreuter, Dong Yang, Jeffrey D Rudolf, Liao-Bin Dong, Ben Shen

**Affiliations:** D epartment of Chemistry, The Herbert Wertheim UF Scripps Institute for Biomedical Innovation & Technology, University of Florida, Jupiter, FL 33458, USA; Skaggs Graduate School of Chemical and Biological Sciences, Scripps Research, Jupiter, FL 33458, USA; D epartment of Chemistry, The Herbert Wertheim UF Scripps Institute for Biomedical Innovation & Technology, University of Florida, Jupiter, FL 33458, USA; D epartment of Chemistry, The Herbert Wertheim UF Scripps Institute for Biomedical Innovation & Technology, University of Florida, Jupiter, FL 33458, USA; D epartment of Chemistry, The Herbert Wertheim UF Scripps Institute for Biomedical Innovation & Technology, University of Florida, Jupiter, FL 33458, USA; D epartment of Chemistry, The Herbert Wertheim UF Scripps Institute for Biomedical Innovation & Technology, University of Florida, Jupiter, FL 33458, USA; Natural Products Discovery Center, The Herbert Wertheim UF Scripps Institute for Biomedical Innovation & Technology, University of Florida, Jupiter, FL 33458, USA; D epartment of Chemistry, The Herbert Wertheim UF Scripps Institute for Biomedical Innovation & Technology, University of Florida, Jupiter, FL 33458, USA; D epartment of Chemistry, The Herbert Wertheim UF Scripps Institute for Biomedical Innovation & Technology, University of Florida, Jupiter, FL 33458, USA; D epartment of Chemistry, The Herbert Wertheim UF Scripps Institute for Biomedical Innovation & Technology, University of Florida, Jupiter, FL 33458, USA; Skaggs Graduate School of Chemical and Biological Sciences, Scripps Research, Jupiter, FL 33458, USA; Natural Products Discovery Center, The Herbert Wertheim UF Scripps Institute for Biomedical Innovation & Technology, University of Florida, Jupiter, FL 33458, USA; Department of Molecular Medicine, The Herbert Wertheim UF Scripps Institute for Biomedical Innovation & Technology, University of Florida, Jupiter, FL 33458, USA

**Keywords:** Biosynthesis, Medium optimization, Pathway engineering, Platencin, Platensilin, Platensimycin

## Abstract

The platensimycin (PTM), platencin (PTN), and platensilin (PTL) family of natural products continues to inspire the discovery of new chemistry, enzymology, and medicine. Engineered production of this emerging family of natural products, however, remains laborious due to the lack of practical systems to manipulate their biosynthesis in the native-producing *Streptomyces platensis* species. Here we report solving this technology gap by implementing a CRISPR-Cas9 system in *S. platensis* CB00739 to develop an expedient method to manipulate the PTM, PTN, and PTL biosynthetic machinery *in vivo*. We showcase the utility of this technology by constructing designer recombinant strains *S. platensis* SB12051, SB12052, and SB12053, which, upon fermentation in the optimized PTM-MS medium, produced PTM, PTN, and PTL with the highest titers at 836 mg L^−1^, 791 mg L^−1^, and 40 mg L^−1^, respectively. Comparative analysis of these resultant recombinant strains also revealed distinct chemistries, catalyzed by PtmT1 and PtmT3, two diterpene synthases that nature has evolved for PTM, PTN, and PTL biosynthesis. The Δ*ptmR1*/Δ*ptmT1*/Δ*ptmT3* triple mutant strain *S. platensis* SB12054 could be envisaged as a platform strain to engineer diterpenoid biosynthesis by introducing varying *ent*-copalyl diphosphate-acting diterpene synthases, taking advantage of its clean metabolite background, ability to support diterpene biosynthesis in high titers, and the promiscuous tailoring biosynthetic machinery.

**One-Sentence Summary:**

Implementation of a CRISPR-Cas9 system in *Streptomyces platensis* CB00739 enabled the construction of a suite of designer recombinant strains for the overproduction of platensimycin, platencin, and platensilin, discovery of new diterpene synthase chemistries, and development of platform strains for future diterpenoid biosynthesis engineering.

## Graphical Abstract

**Graphical Abstract:**
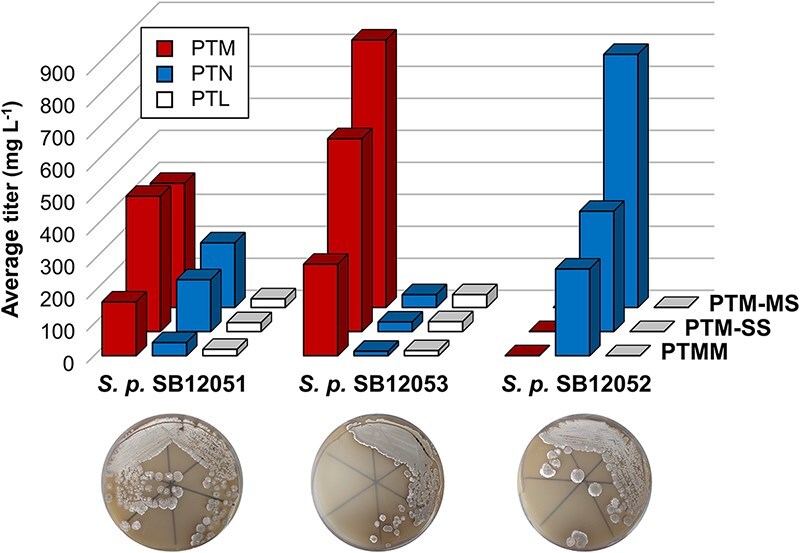

## Introduction

**Fig. 1.**
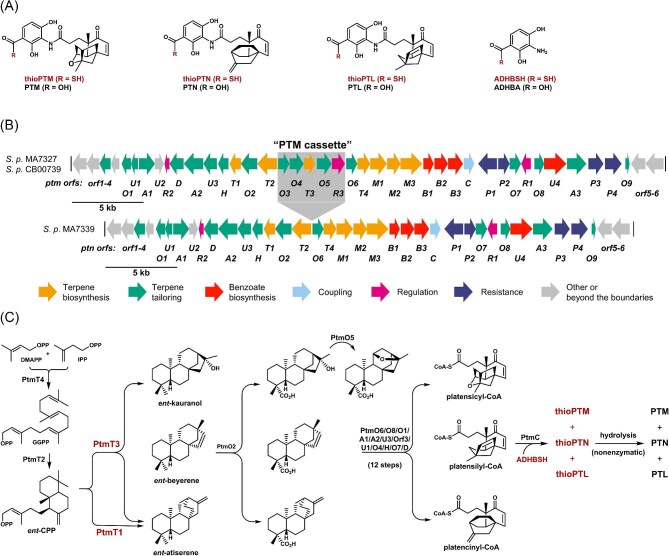
Platensimycin (PTM), platencin (PTN), and platensilin (PTL) structures and biosynthesis. (A) The structures of PTM, PTN, and PTL, as well as the key biosynthetic intermediate 3-amino-2,4-dihydroxybenzoic acid (ADHBA), and their nascent thioacid congeners. (B) Genetic organization of the *ptm* gene cluster from *S. platensis* MA7327 and *S. platensis* CB00739 encoding the production of PTM, PTN, and PTN, and the *ptn* gene cluster from *S. platensis* MA7339 encoding the production of PTN only, with the PTM cassette shaded in gray to highlight the difference between the two gene clusters. (C) PTM, PTN, and PTL biosynthesis featuring two diterpene synthases, PtmT1 and PtmT3, that channel the common precursor *ent*-copalyl pyrophosphate (*ent*-CPP) into three distinct scaffolds *ent*-kauranol, *ent*-beyerene, and *ent*-atiserene, and a promiscuous tailoring biosynthetic machinery that acts on all three scaffolds to account for structural diversity of this family of natural products.

Platensimycin (PTM), platencin (PTN), and platensilin (PTL) are members of an emerging family of bacterial natural products that have been intensively pursued as promising antibacterial and antidiabetic drug leads (Fig. 1A) (Rudolf et al., 2017; Zheng et al., 2021; Wang et al., 2006, 2007). PTM was first isolated from *Streptomyces platensis* MA7327 (Singh et al., 2006; Wang et al., 2006), and PTN was isolated from *S. platensis* MA7339 (Wang et al., 2007). Subsequent discovery of PTN production in *S. platensis* MA7327 (Herath et al., 2008) and comparative analysis of the *ptm* biosynthetic gene cluster (BGC) from *S. platensis* MA7327 and the *ptn* BGC from *S. platensis* MA7339 revealed *S. platensis* MA7327 as a PTM and PTN dual producer (Fig. 1B) (Smanski et al., 2009, 2011). Motivated by the search for alternative producers with enhanced genetic amenability to expedite *in vivo* manipulation of PTM and PTN biosynthesis, we identified six alternative PTM and PTN dual producers by mining the Actinobacteria strain collection of the Natural Products Discovery Center at the Wertheim UF Scripps Institute (Hindra et al., 2014). *S. platensis* CB00739, one of the six alternative producers, was subsequently developed into a platform strain for PTM and PTN biosynthesis. *S. platensis* SB12029, a recombinant strain of *S. platensis* CB00739, massively overproducing PTM and PTN (Hindra et al., 2014; Shi et al., 2016), enabled (1) the discovery of the minor product PTL (Zheng et al., 2021) and (2) the revelation of thioplatensimycin (thioPTM), thioplatencin (thioPTN), and thioplatensilin (thioPTL) as the nascent final products of the PTM, PTN, and PTL biosynthetic machinery (Fig. 1A, C) (Dong et al., 2016, 2018; Zheng et al., 2021).

Consisting of a varying diterpenoid-derived ketolide moiety linked via a flexible propionamide chain to 3-amino-2,4-dihydroxybenzoic acid, the PTM, PTN, and PTL family of natural products continues to inspire the discovery of novel biosynthetic chemistry and enzymology (Fig. 1A) (Rudolf et al., 2017; Zheng et al., 2021). Among the many characteristic features of the PTM, PTN, and PTL biosynthetic machinery are (1) two diterpene synthases, PtmT3 and PtmT1, that partition a common diterpene precursor *ent*-copalyl diphosphate (*ent*-CPP) into three distinct diterpene scaffolds, for example, *ent*-kauranol, *ent*-atiserene, and *ent*-beyerene, and (2) a common tailoring biosynthetic machinery, consisting of minimally 14 enzymes, that acts on all three scaffolds parallelly to afford thioPTM, thioPTN, and thioPTL as the nascent products, which undergo nonenzymatic hydrolysis into PTM, PTM, and PTL during isolation (Fig. 1B, C). The tailoring biosynthetic machinery displays remarkable substrate promiscuity, a biosynthetic logic that serves as an inspiration to access natural product diversity and engineer designer analogues by combinatorial biosynthesis and synthetic biology (Smanski et al., 2016; Teijaro et al., 2019).

PTM, PTN, and PTL biosynthesis and production have benefited greatly from a suite of engineered overproducers (Table 1). The first generation PTM and PTN overproducers were engineered in *S. platensis* MA7327 or *S. platensis* MA7339 by replacing the pathway-specific negative transcriptional regulator *ptmR1* or *ptnR1* with the apramycin resistance gene *aac(3)IV*, as exemplified by *S. platensis* SB12001 and SB12002, or *S. platensis* SB12600, respectively (Smanski et al., 2009; Yu et al., 2010). These Δ*ptmR1* or Δ*ptnR1* recombinant strains overproduce PTM and PTN, but they sporulate poorly and are recalcitrant to further *in vivo* manipulation. The second-generation overproducers were engineered in *S. platensis* CB00739 similarly by replacing *ptmR1* with *aac(3)IV* but followed by removing the *aac(3)IV* cassette via a λ-RED and FLP/FRT-mediated site-specific recombination, as exemplified by *S. platensis* SB12029 (Gust et al., 2003, 2004; Rudolf et al., 2015). While *S. platensis* SB12029 overproduces PTM and PTN, the removal of *aac(3)IV* via the λ-RED and FLP/FRT-mediated method required weeks of tedious screening, making the generation of multiple mutations impractically laborious; it also leaves an 81-bp scar within each of the targeted genes, the effect of which on functional expression of the engineered BGCs is uncertain. As a result, no markerless double mutant has been constructed in *S. platensis* CB00739 to date. Expression of the *ptn* BGC from *S. platensis* MA7339 in selected model *Streptomyces* hosts has also been explored to circumvent the challenges in manipulating PTM and PTN biosynthesis in their native producers, as exemplified by *S. lividans* SB12606 that harbors the *ptn* BGC with the Δ*ptnR1* mutation in *Streptomyces lividans* K4-114. However, *S. lividans* SB12606 produces PTN at very low titers (Smanski et al., 2012, 2016).

**Table 1.** Development of PTM, PTN, and PTL Overproducers by Biosynthetic Pathway Engineering and Fermentation Medium Optimization of *Streptomyces platensis* MA7327, MA7339, CB00739, and Their Recombinant Variants^a^,^b^

				Titer (mg L^−1^)			
	*S. platensis* strain	Genotype	Medium	PTM	PTN	PTL	Reference
**PTM, PTN, & PTL overproducer (Δ*ptmR1*)**	MA7327	Wild-type	PTMM	15.1 ± 18.2	2.5 ± 0.7	ND	Smanski et al., 2009
	SB12001	MA7237 (Δ*ptmR1::apr^R^*)	PTMM	157 ± 22	255 ± 30	ND	Smanski et al., 2009
	SB12002	MA7237 (Δ*ptmR1::apr^R^*)	PTMM	323 ± 29	51 ± 9.2	ND	Smanski et al., 2009
	CB00739	Wild-type	PTMM	1.0 ± 0.3	1.6 ± 0.2	ND	Hindra et al, 2014
	SB12026	CB00739 (Δ*ptmR1::apr^R^*)	PTMM	310 ± 12	170 ± 6	ND	Hindra et al., 2014
	SB12029	CB00739 (Δ*ptmR1::*18-bp scar)	PTMM	195 ± 24	49 ± 5	16 ± 2	Rudolf et al., 2015; this study
			PTM-SS	437 ± 47	151 ± 23	24 ± 3	This study
	**SB12051**	**CB00739 (Δ*ptmR1*)**	PTMM	168 ± 30	41 ± 7	20 ± 5	This study
			PTM-SS	423 ± 75	162 ± 29	28 ± 6	This study
			**PTM-MS**	**388 ± 18**	**202 ± 24**	**27 ± 4**	This study
**PTM overproducer (Δ*ptmR1/*Δ*ptmT1*)**	**SB12053**	**CB00739 (Δ*ptmR1/*Δ*ptmT1*)**	PTMM	286 ± 29	14 ± 1	16 ± 1	This study
			PTM-SS	603 ± 77	30 ± 2	31 ± 4	This study
			**PTM-MS**	**836 ± 96**	**40 ± 10**	**40 ± 5**	This study
**PTN overproducer (Δ*ptnR1*) or (Δ*ptmR1/*Δ*ptmT3*)**	MA7339	Wild-type	PTMM	–	0.05 ± 0.04	–	Yu et al, 2010
	SB12600	MA7339 (Δ*ptmR1*)	PTMM	–	22 ± 3.0	–	Yu et al., 2010
	**SB12052**	**CB00739 (Δ*ptmR1/*Δ*ptmT3*)**	PTMM	0.0	271 ± 24	0.0	This study
			PTM-SS		376 ± 56	0.0	This study
			**PTM-MS**	**0.0**	**791 ± 119**	**0.0**	This study

a PTL was not discovered until 2021 (Zheng et al., 2021), and PTL titers therefore were not determined in the earlier studies. ND, not determined.

b Boldface highlights the engineered strains that afford the highest PTM, PTN, and PTL titers when fermented under the optimized PTM-MS medium.

Systems based on clustered regularly interspaced short palindromic repeats (CRISPR) and CRISPR-associated (Cas) proteins have been recently introduced for genetic manipulation in selected *Streptomyces* species that allow for fast and precise genome editing (Cobb et al., 2015; Huang et al., 2015; Liu et al., 2020; Tong et al., 2015, 2019; Zeng et al., 2015). Implementation of a CRISPR/Cas9 system in *S. platensis* CB00739 would overcome the limitations of the λ-RED and FLP/FRT-mediated methods and facilitate efficient and precise *in vivo* manipulation of the PTM, PTN, and PTL biosynthetic machinery in *S. platensis*. Herein, we report the CRISPR/Cas9-mediated engineering of *S. platensis* CB00739 to generate recombinant strains that overproduce PTM (*S. platensis* SB12053), PTN (*S. platensis* SB12052), or all three metabolites of PTM, PTN, and PTL (*S. platensis* SB12051). Medium and fermentation optimization further highlight the utility of these engineered strains for PTM, PTN, and PTL production. The power of this CRISPR/Cas9-based genetic engineering strategy is finally showcased by the construction of the first triple mutant strain, *S. platensis* SB12054, setting the stage to exploit the PTM, PTN, and PTL biosynthetic machinery to engineer designer analogues for this emerging family of natural products.

## Materials and Methods

### Bacterial Strains, Plasmids, and Chemicals

Lists of oligonucleotides, plasmids, and bacterial strains used in this study are provided (Supplementary Tables S1, S2). Oligonucleotides were purchased from Sigma Aldrich (Boston, MA, USA) and Integrated DNA Technologies (Coralville, IA, USA). Kits from Omega Bio-Tek (Norcross, GA, USA) were used for gel extraction of DNA and plasmid preparation. Restriction endonucleases, Q5 high-fidelity DNA polymerase, Gibson Assembly Master Mix, and T4 DNA ligase were all purchased from New England Biolabs (Ipswich, MA, USA) and used according to the manufacturer's instructions. The DIG High Prime DNA Labeling and Detection Starter Kit I from Roche (Basel, Switzerland) was used for Southern blot analyses. Other chemicals and components for culture media were purchased from standard commercial sources and used as is. DNA Sanger sequencing was performed by Genewiz (South Plainfield, NJ, USA).

### General Experimental Procedures

*Escherichia coli* strains were cultured in lysogeny broth with appropriate antibiotics (Sambrook & Russel, 2001). The wild-type and recombinant strains of *S. platensis* CB00739 were grown at 28°C on solid ISP2 and ISP4 medium for sporulation (Shirling & Gottlieb, 1966) or liquid tryptic soy broth for growth of mycelium, seed cultures, and isolation of genomic DNA (gDNA) with appropriate antibiotics. High-performance liquid chromatography-mass spectrometry (HPLC-MS) was performed using an Agilent (Santa Clara, CA, USA) 1260 Infinity LC coupled to a 6230 TOF (high-resolution electrospray ioinization) equipped with an Agilent Poroshell 120 EC-C18 column (4.6 × 50 mm, 2.7 μm).

### Genetic Engineering of *S. Platensis* CB00739 and Mutant Variants Using CRISPR/Cas9

Identification of potential 20-nt single guide RNA (sgRNA) target sequences in the *S. platensis* CB00739 chromosome was carried out using CasOT (Xiao et al., 2014). Candidate sequences with a predicted low probability of off-target effects were selected for construction of the pCRISPomyces-2-based editing plasmids, which followed the standard protocol (Cobb et al., 2015). In brief, for each target gene, a set of paired oligonucleotides representing the selected sgRNA sequence were annealed and inserted into pCRISPomyces-2 via Golden Gate assembly. Simultaneously, two ∼2-kb homology arms, one from each end of the targeted gene, were amplified via polymerase chain reaction (PCR) from the gDNA of *S. platensis* CB00739. The sgRNA-containing plasmids were then digested by *Xba*I and assembled with the two homology arms in a three-piece Gibson assembly to afford the final disruption plasmids pBS12129 (Δ*ptmR1*), pBS12130 (Δ*ptmT3*), and pBS12131 (Δ*ptmT1*).

The disruption plasmids were introduced into *S. platensis* CB00739 and its mutant variants via intergeneric conjugation from the methylation-deficient *E. coli* strain ET12567/pUZ8002 (Hopwood et al., 2000). Exconjugants were restruck onto ISP2 plates supplemented with 30 μg mL^−1^ apramycin and grown at 28°C for 3 days. Single colonies were grown in TSB medium for gDNA isolation. The target region was PCR-amplified with primers binding outside of the target region, and the products were analyzed via gel electrophoresis. Colonies that showed a pattern reflecting the desired mutation were selected for curing of the editing plasmid. The colonies were first subjected to two rounds of growth on nonselective ISP2 at 37°C before being replica-plated on both selective and nonselective ISP2 plates. Colonies showing the desired sensitivity to apramycin were grown in TSB to isolate gDNA. The genotypes of the recombinant strains were confirmed via diagnostic PCR, Southern blot analysis, and Sanger sequencing (Supplementary Figs. S1–S3). While the Δ*ptmR1* strain *of S. platensis* SB12051 was constructed by deleting *ptmR1* in *S. platensis* CB00739, the double mutants of *S. platensis* SB12052 (Δ*ptmR1* and Δ*ptmT3*) and *S. platensis* SB12053 (Δ*ptmR1* and Δ*ptmT1*) were constructed by deleting *ptmT3* and *ptmT1* in *S. platensis* SB12051, respectively. The triple mutant in *S. platensis* SB12054 (Δ*ptmR1*, Δ*ptmT3*, and Δ*ptmT1*) was constructed by deleting *ptmT1* from *S. platensis* SB12052.

### Fermentation and HPLC-MS Analysis of *S. Platensis* SB12051, SB12052, SB12053, and SB12054

Fermentation of *S. platensis* SB12051, SB12052, SB12053, and SB12054, with *S. platensis* CB00739 as a control, followed previous protocols (Rudolf et al., 2015; Smanski et al., 2009; Zheng et al., 2021). Briefly, spores of the *S. platensis* strains of interest were inoculated into TSB medium and cultured at 28°C and 250 rpm for 2 days to yield a seed culture. The original production medium, known as PTM medium and renamed PTMM here for clarity, consisted of 40 g L^−1^ dextrin, 40 g L^−1^ α-lactose, and 5 g L^−1^ yeast extract, pH 7.0 (Smanski et al., 2009). In 250 mL baffled flasks, 50 mL of PTMM was charged with 3% (w/v) Amberlite XAD-16 resin (Sigma Aldrich, Boston, MA, USA), autoclaved, and inoculated with 4% (v/v) seed culture. The fermentation cultures were incubated at 28°C and 250 rpm for 7 days. Resin was separated from mycelium by diluting the culture with water, allowing the resin to settle, and decanting cell debris. This was repeated until no visible debris remained. The resin was extracted with methanol (3 × 10 mL). An aliquot of the extract was diluted 1:10 in methanol and centrifuged prior to analysis. Liquid chromatography for HPLC-MS analysis was performed using an 18-min solvent gradient from 5% to 95% methanol in water containing 0.1% formic acid at a flow rate of 0.4 mL min^−1^. The peak area at 254 nm was used to quantify PTM, PTL, and PTN titers using absorbance coefficients obtained from standard calibration curves generated using pure compounds. Reported titers represent mean values from at least three independent biological replicates.

### Single-Factor Medium Optimization for PTL, PTM, and PTN Production

The starting optimized medium “PTM-SS,” consisting of 70 g L^−1^ soluble starch, 15 g L^−1^ soybean flour, 5 g L^−1^ 3-(N-morpholino)propanesulfonic acid (MOPS), 15 mg L^−1^ MnCl_2_·4H_2_O, and 30 mg L^−1^ (NH_4_)_6_Mo_7_O_24_·4H_2_O, pH 7.2, was adopted from a previous study (Shi et al., 2016). During medium optimization, the inorganic salts and pH were unchanged. The concentration of carbon sources and nitrogen sources was maintained at 70 g L^−1^ and 15 g L^−1^, respectively. The effects of carbon sources, varying from soluble starch, dextrin, α-lactose, glycerin, α-sucrose, to α-maltose, and nitrogen sources, varying from soybean flour, yeast extract, beef extract, cottonseed meal, peptone, and soytone, on PTM, PTN, and PTL titers were examined (Supplementary Tables S5, S6). The final optimized medium “PTM-MS” consisted of 70 g L^−1^ α-maltose, 15 g L^−1^ soybean flour, 5 g L^−1^ MOPS, 15 mg L^−1^ MnCl_2_·4H_2_O, and 30 mg L^−1^ (NH_4_)_6_Mo_7_O_24_·4H_2_O, pH 7.2.

## Results and Discussion

### Engineering of PTM, PTN, PTL Biosynthesis by Applying CRISPR-Cas9 System to *S. Platensis* CB00739

**Fig. 2.**
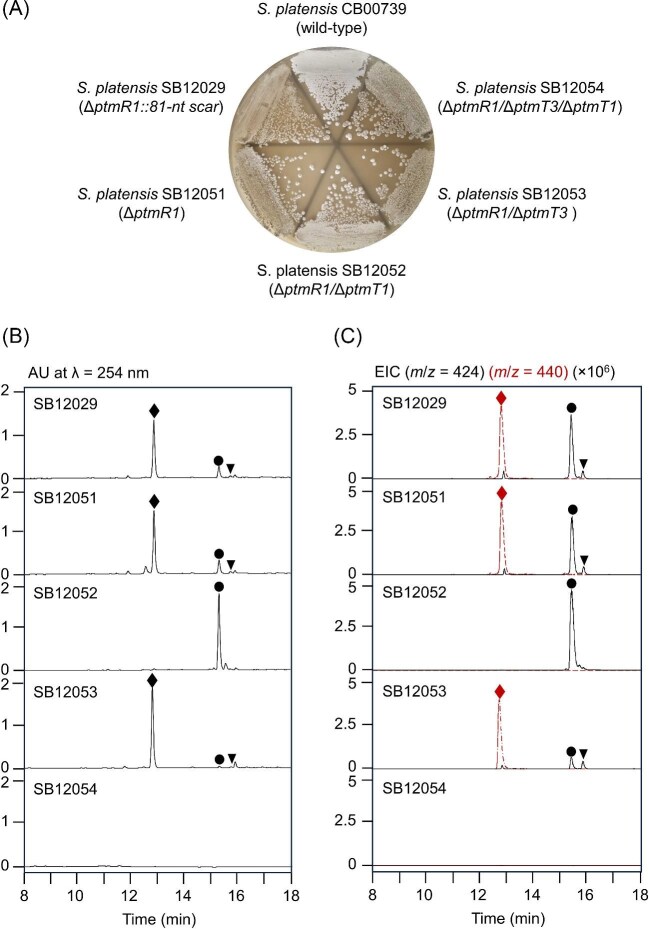
Engineered *S. platensis* recombinant strains that overproduce PTM, PTN, and PTN. (A) Morphology of the *S. platensis* CB00739 wild-type strain in comparison with the CRISPR-Cas9-enabled recombinant strains *S. platensis* SB12051, SB12052, SB12053, and SB12054 and λ-RED and FLP/FRT-mediated recombinant strain *S. platensis* SB12029, all of which sporulated well on several media as exemplified with ISP4. Metabolite profiles, following fermentation in PTMM medium, of *S. platensis* SB12051, SB12052, SB12053, and SB12054 in comparison with *S. platensis* CB00739 upon HPLC-MS analysis with (B) UV detection at 254 nm or (C) negative-mode electrospray ionization mass spectrometry and ion extraction at *m*/*z *= 424 (black solid line, the expected mass of PTN and PTL) and *m*/*z *= 440 (dashed red line, the expected mass of PTM). Also see Supplementary Fig. S4 for full-length chromatograms with authentic standards. PTM (♦), PTN (●), and PTL (▼).

The feasibility of applying the CRISPR/Cas9 system to engineer the PTM, PTN, and PTL biosynthetic machinery was demonstrated in *S. platensis* CB00739 by constructing the scarless Δ*ptmR1* mutant strain *S. platensis* SB12051. Introduction of the pCRISPomyces-2-based *ptmR1*-targeting plasmid pBS12129 and subsequent curing of the plasmid yielded the scarless Δ*ptmR1* mutant strain *S. platensis* SB12051 (Supplementary Fig. S1). This strategy avoided the laborious process of inserting and screening for removal of the *acc(3)IV* resistance cassette via the λ-RED and FLP/FRT-mediated methods used previously to construct the Δ*ptmR1* mutant strain *S. platensis* SB12029 (Rudolf et al., 2015). The growth phenotype of *S. platensis* SB12051 matched that of *S. platensis* SB12029, and most importantly, *S. platensis* SB12051 displayed dense sporulation, implying that it avoids the drawback of the overproducers engineered previously, whose poor sporulation phenotype has hampered further genetic manipulation (Fig. 2A) (Hindra et al., 2014; Smanski et al., 2009). *S. platensis* SB12051 was fermented under established fermentation conditions with *S. platensis* SB12029 as a control, and crude extracts of the fermentation cultures were subjected to HPLC-MS analysis to compare metabolite profiles (Hindra et al., 2014; Smanski et al., 2009). *S. platensis* SB12051 overproduced PTM (168 mg L^−1^), PTN (41 mg L^−1^), and PTL (20 mg L^−1^), comparable to those of *S. platensis* SB12029 (Fig. 2B, C, Table 1). Taken together, these findings confirm the compatibility of the CRISPR/Cas9 system with *S. platensis* CB00739 and set the stage to further manipulate PTM, PTN, and PTL biosynthesis by iterative application of the CRISPR/Cas9 system to the engineered overproducer *S. platensis* SB12051.

### Engineering of a PTN Overproducer by Deleting *ptmT3* in *S. Platensis* SB12051

As the two diterpene synthases PtmT3 and PtmT1 partition the common precursor *ent*-CPP into the three PTM, PTN, and PTL scaffolds in their biosynthesis (Smanski et al., 2011; Zheng et al., 2021), we first applied the CRISPR/Cas9 system to delete *ptmT3* in *S. platensis* SB12051, affording the Δ*ptmR1*/Δ*ptmT3* double mutant strain *S. platensis* SB12052 (Supplementary Fig. S2). *S. platensis* SB12052 exhibited similar growth characteristics as *S. platensis* SB12051, including dense sporulation (Fig. 2A). *S. platensis* SB12052 was similarly fermented under the established conditions, with *S. platensis* SB12051 as a control, and crude extracts of the fermentation cultures were analyzed by HPLC-MS for changes in the metabolite profile (Hindra et al., 2014; Smanski et al., 2009). Production of PTM and PTL was completely abolished in *S. platensis* SB12052, with a concomitant increase (>6-fold) in PTN titer (271 mg L^−1^) (Fig. 2B, C, Table 1). The fact that *S. platensis* SB12052 produces PTN with titers that are >12-fold higher than *S. platensis* SB12600 engineered from the PTN producer *S. platensis* MA7339 (Yu et al., 2010) further supports *S. platensis* SB12051 as a superior strain for engineered production of the PTM, PTN, and PTL family of natural products (Table 1). Consistent with the role of PtmT1 as a dedicated *ent*-atiserene synthase (Smanski et al., 2011), together with the finding that the PTN titer in *S. platensis* SB12052 is comparable to the overall titers of PTM, PTN, and PTL in *S. platensis* SB12051, these findings also reveal that the metabolic flux of the PTM, PTN, and PTL biosynthetic machinery could be exploited to engineer designer analogues by manipulating the gatekeeping diterpene synthases as exemplified by PtmT1 and PtmT3, taking advantage of the inherent substrate promiscuity of the tailoring biosynthetic machinery (Fig. 1C) (Dong et al., 2019; Wang et al., 2018; Zheng et al., 2021).

### Engineering of a PTM Overproducer by Deleting *ptmT1* in *S. Platensis* SB12051

Complementary to the Δ*ptmR1*/Δ*ptmT3* double mutant strain *S. platensis* SB12052 that overproduced PTN, we next applied the CRISPR/Cas9 system to delete *ptmT1* in *S. platensis* SB12051, affording the Δ*ptmR1*/Δ*ptmT1* double mutant strain *S. platensis* SB12053 (Supplementary Fig. S3). Like *S. platensis* SB12051 and *S. platensis* SB12052, *S. platensis* SB12053 also grew and sporulated well (Fig. 2A). Upon fermentation of *S. platensis* SB12053 under the established conditions, the crude extracts of the fermentation cultures were analyzed by HPLC-MS for changes in the metabolite profile (Hindra et al., 2014; Smanski et al., 2009). As expected, *S. platensis* SB12053 overproduced PTM with a significantly increased titer (286 mg L^−1^), but PTL titer (16 mg L^−1^) remained largely unchanged, and most surprisingly, it still produced PTN, albeit with a significantly reduced titer (14 mg L^−1^) (Fig. 2B, C, Table 1). The latter observation revealed that PtmT3 is capable of producing *ent*-atiserene as a minor product, a well-known property of plant *ent*-kauranol synthases (Xu et al., 2007). Nature has apparently evolved two distinct chemistries, that is, PtmT1 and PtmT3, for PTN biosynthesis (Fig. 1B, C). This subtlety of the PTM, PTN, and PTL biosynthetic machinery escaped detection in the Δ*ptmT3* mutant strain *S. platensis* SB12008 made previously from the original *S. platensis* MA7327 due to low overall titers (Smanski et al., 2011), underscoring the advantage of the recombinant strains enabled by the CRISPR/Cas9-based system in the current study for future investigation of PTM, PTN, and PTL biosynthesis and engineered production.

### Engineering of a Platform Strain for Diterpenoid Biosynthesis and Production by Deleting Both *ptmT1* and *ptmT3* in *S. Platensis* SB12051

We lastly confirmed that PtmT1 and PtmT3 control the channeling of *ent*-CPP into PTM, PTN, and PTL biosynthesis in *S. platensis* by applying the CRISPR/Cas9 system to delete both *ptmT1* and *ptmT3* from *S. platensis* SB12052, affording the Δ*ptmR1*/Δ*ptmT1/*Δ*ptmT3* triple mutant strain *S. platensis* SB12054 (Fig. 1B, C, and Supplementary Fig. S3). *S. platensis* SB12054 exhibited the same growth and sporulation characteristics as the other CRISPR/Cas9-enabled recombinant strains (Fig. 2A). Fermentation of *S. platensis* SB12054 under the established conditions, followed by HPLC-MS analysis of the crude extracts of the fermentation cultures (Hindra et al., 2014; Smanski et al., 2009), demonstrated complete abolishment of PTM, PTN, and PTL production (Fig. 2B, C). Notably, *S. platensis* SB12054 is the first triply mutated recombinant *S. platensis* strain engineered to date, highlighting the power of the CRISPR/Cas9 system for genetic manipulation of the PTM, PTN, and PTL biosynthetic machinery. Complementary to the bottom-up strategy of accessing diterpenoid structural diversity by introducing varying diterpene synthases into a suite of engineered *E. coli* chassis strains (Cyr et al., 2007), *S. platensis* SB12054 could be similarly exploited as a platform strain by introducing varying diterpene synthases to engineer diterpenoid biosynthesis (Fig. 1B, C).

### Medium Optimization of *S. Platensis* SB12051, SB12052, and SB12053 for PTM, PTN, and PTL Production

**Fig. 3.**
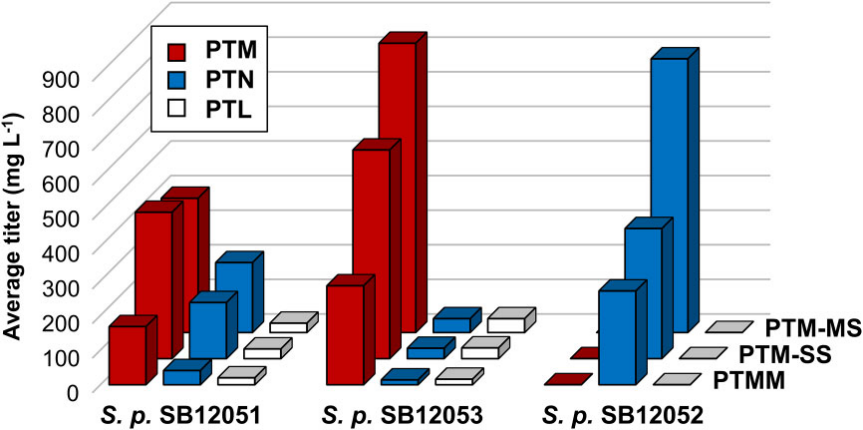
PTM, PTN, and PTL titers from fermentation of *S. platensis* SB12051, SB12052, and SB12053 in three media highlighting PTM-MS as the optimized medium for production. Bar heights reflect the mean titers of at least three biological replicates. See Table 1 for standard deviation.

We finally further improved PTM, PTN, and PTL production titers by subjecting *S. platensis* SB12051, SB12052, and SB12053 to fermentation medium optimization (Fig. 3, Table 1). Most of the published studies on PTM, PTN, and PTL biosynthesis and production to date used the production medium known as PTM medium (herein renamed as PTMM), which was developed for the *S. platensis* MA7327 and MA7339 wild-type, as well as their Δ*ptmR1* or Δ*ptnR1* mutant strains (Smanski et al., 2009; Yu et al., 2010). An optimized production medium, known as “PTM-SS” medium (Shi et al., 2016), was also developed by systematically varying the carbon and nitrogen sources, as well as the inorganic salts, for *S. platensis* SB12026 to produce PTM on a pilot scale, affording an impressive PTM titer of 1,560 mg L^−1^ in 15-L fermenters (Shi et al., 2016). We therefore first subjected *S. platensis* SB12051, SB12052, and SB12053, together with *S. platensis* SB12029 as a control, to fermentation in the “PTM-SS” medium. Significant increases in PTM, PTN, and PTL titers, varying between 1.5 and 2-fold, were observed across all the strains tested (Fig. 3, Table 1). Encouraged by these findings, we next focused on fine-tuning the carbon and nitrogen sources for their effect on production by *S. platensis* SB12052 and SB12053 while keeping the inorganic salts and pH of the “PTM-SS” medium unchanged. Among the five carbon and six nitrogen sources examined, the highest titers for both strains were enabled by the combination of α-maltose and soybean flour as the preferred carbon and nitrogen sources (Supplementary Tables S3, S4). We named this optimized production medium as “PTM-MS” medium and finally demonstrated further improved production of PTM, PTN, and PTL in shake flasks using the “PTM-MS” medium. *S. platensis* SB12052 produced PTN exclusively with titers reaching 791 mg L^−1^, while *S. platensis* SB12053 produced PTM as the predominant product, with titers reaching 836 mg L^−1^, and PTN and PTL as minor products, with titers reaching 40 mg L^−1^ each, respectively (Fig. 3, Table 1). These findings highlight the power of combining contemporary metabolic pathway engineering strategies with traditional medium and fermentation optimization methods for strain development and titer improvement, setting the stage for future production of the PTM, PTN, and PTL family of natural products, as well as their engineered analogues, at pilot scales for further development as promising antibacterial and antidiabetic drug leads.

## Conclusions

Biosynthesis and engineering of the PTM, PTN, and PTL family of natural products have been hampered by the lack of practical systems to manipulate their biosynthesis in the native-producing *S. platensis* species (Rudolf et al., 2017; Zheng et al., 2021). Expression of the BGC encoding PTN biosynthesis in model *Streptomyces* hosts has fallen short of overcoming the challenge, producing PTN only at a very low titer (<2.0 mg L^−1^) (Smanski et al., 2012). We have now solved this technology gap by implementing the CRISPR-Cas9 system in *S. platensis* CB00739 and developed an expedient method to manipulate the PTM, PTN, and PTL biosynthetic machinery *in vivo* (Fig. 2). We showcased the utility of this technology by constructing designer recombinant strains *S. platensis* SB12051, SB12052, and SB12053 which, upon fermentation in the optimized PTM-MS medium, produced PTM, PTN, and PTL with the highest titers at 836 mg L^−1^, 791 mg L^−1^, and 40 mg L^−1^, respectively (Table 1). Comparative analysis of PTM, PTN, and PTL biosynthesis between *S. platensis* SB12052 (Δ*ptmR1*/Δ*ptmT3*), SB12053 (Δ*ptmR1*/Δ*ptmT1*), and SB12054 (Δ*ptmR1*/Δ*ptmT1*/ΔptmT3) also revealed two distinct chemistries, catalyzed by the two diterpene synthases PtmT1 and PtmT3, that nature has evolved for PTM, PTN, and PTL biosynthesis (Fig. 1B, C). Further, *S. platensis* SB12054 could be exploited as a platform strain by introducing varying *ent*-CPP-acting diterpene synthases to engineer diterpenoid biosynthesis, taking advantage of its clean metabolite background, ability to support diterpene biosynthesis in high titers, and the promiscuous tailoring biosynthetic machinery that can transform the nascent diterpene scaffolds to further enrich structural diversity.

## Supplementary Material

kuae003_Supplemental_File
