# Supplementary material for: Development of platensimycin, platencin, and platensilin overproducers by biosynthetic pathway engineering and fermentation medium optimization
Source: J Ind Microbiol Biotechnol. 2024 Jan 23;51:kuae003. doi: 10.1093/jimb/kuae003 (PMC10847714; doi:10.1093/jimb/kuae003)
Supplement: kuae003_Supplemental_File [file kuae003_supplemental_file.doc]

**Development of platensimycin, platencin, and platensilin overproducers by biosynthetic pathway engineering and fermentation medium optimization**

Lucas L. Fluegel,^1,2,#^ Ming-Rong Deng,^1,#^ Ping Su^1,#^ Edward Kalkreuter,^1^ Dong Yang,^1,3^ Jeffrey D. Rudolf,^1^ Liao-Bin Dong,^1^ and Ben Shen^1,2,3,4*^

^1^Department of Chemistry, The Herbert Wertheim UF Scripps Institute for Biomedical Innovation & Technology, University of Florida, Jupiter, Florida 33458, United States

^2^Skaggs Graduate School of Chemical and Biological Sciences, Scripps Research, Jupiter, Florida 33458, United States

^3^Natural Products Discovery Center, The Herbert Wertheim UF Scripps Institute for Biomedical Innovation & Technology, University of Florida, Jupiter, Florida 33458, United States

^4^Department of Molecular Medicine, The Herbert Wertheim UF Scripps Institute for Biomedical Innovation & Technology, University of Florida, Jupiter, Florida 33458, United States

^#^These authors contributed equally

*Corresponding author: E-mail: [shenb@scripps.edu](mailto:shenb@scripps.edu)

**Table of Contents**

**Supplementary Tables**

Table S1. List of oligonucleotides used in this study S2

Table S2. List of plasmids and bacterial strains used in this study S3

Table S3. Single-factor medium optimization for *S. platensis* SB12052 S4

Table S4. Single-factor medium optimization for *S. platensis* SB12053 S4

**Supplementary Figures**

Figure S1. Construction and genotype verification of *S. platensis* SB12051 S5

Figure S2. Construction and genotype verification of *S. platensis* SB12052 S6

Figure S3. Construction and genotype verification of *S. platensis* SB12053 and SB12054 S7

Figure S4. HPLC-MS profiles of engineered *S. platensis* recombinant strain fermentations S9

**Supplementary References** S11

**Table S1.** List of oligonucleotides used in this study

| Primer | Nucleotide Sequence (5'-3') | Description |
| --- | --- | --- |
| *ptmR1*-sgRNA-F1 | ACGCGAGAAGGAATTGAATGCGAG | Insert gRNA spacer sequence targeting *ptmR1* into pCRISPomyces-2 |
| *ptmR1*-sgRNA-R1 | AAACCTCGCATTCAATTCCTTCTC | Insert gRNA spacer sequence targeting *ptmR1* into pCRISPomyces-2 |
| *ptmR1*-up-F | TCGGTTGCCGCCGGGCGTTTTTTATGGACTCGCGCTGTCCCGCC | Amplify the 2 kb upstream region flanking *ptmR1* |
| *ptmR1*-up-R | TAGGAGGAAGCGGCCGCCCGGCCCGAGCGGTCC | Amplify the 2 kb upstream region flanking *ptmR1* |
| *ptmR1*-down-F | GCTCGGGCCGGGCGGCCGCTTCCTCCTAGGTGG | Amplify the 2 kb downstream region flanking *ptmR1* |
| *ptmR1*-down-R | GCGGCCTTTTTACGGTTCCTGGCCTTGGGGCGGCTCGACCCAG | Amplify the 2 kb downstream region flanking *ptmR1* |
| *ptmR1*-screen-F | GTGCACCACGTTCGGGGAGCAC | Screen the Δ*ptmR1* mutant |
| *ptmR1*-screen-R | GTGCAGGAAGACATCCGTCTCCGCG | Screen the Δ*ptmR1* mutant |
| *ptmR1*-Southern-F | ATGTGGACCGTCTGTGATCG | Amplify the Southern probe for *ptmR1* |
| *ptmR1*-Southern-R | AGGAGGTGTTGATGACGCAG | Amplify the Southern probe for *ptmR1* |
| *ptmT3*-sgRNA-F1 | ACGCGTCCGCGCTGTCCAGTAAGT | Insert gRNA spacer sequence targeting *ptmT3* into pCRISPomyces-2 |
| *ptmT3*-sgRNA-R1 | AAACACTTACTGGACAGCGCGGAC | Insert gRNA spacer sequence targeting *ptmT3* into pCRISPomyces-2 |
| *ptmT3*-up-F | TCGGTTGCCGCCGGGCGTTTTTTATGGCGTTGGTGGAGTTGTTC | Amplify the 2 kb upstream region flanking *ptmT3* |
| *ptmT3*-up-R | GTGCGAACACGCTTCATTCCTGCTTCCTCACG | Amplify the 2 kb upstream region flanking *ptmT3* |
| *ptmT3*-down-F | GGAAGCAGGAATGAAGCGTGTTCGCACCGTCCCGT | Amplify the 2 kb downstream region flanking *ptmT3* |
| *ptmT3*-down-R | GCGGCCTTTTTACGGTTCCTGGCCTTCGTCGAAGGCCGTCAGC | Amplify the 2 kb downstream region flanking *ptmT3* |
| *ptmT3*-screen-F | CGCAACTGTTGTTGTCGTTCGTGGTG | Screen the Δ*ptmT3* mutant |
| *ptmT3*-screen-R | GGCACGAACGCCAGATTGCC | Screen the Δ*ptmT3* mutant |
| *ptmT3*-Southern-F | TACCTCACCATCCCGCAGTT | Amplify the Southern probe for *ptmT3* |
| *ptmT3*-Southern-R | CCTTGACGAACAGCCTCTCC | Amplify the Southern probe for *ptmT3* |
| *ptmT1*-sgRNA-F | ACGCCGTGATGAGGACGGGAGGGG | Insert gRNA spacer sequence targeting *ptmT1* into pCRISPomyces-2 |
| *ptmT1*-sgRNA-R | AAACCCCCTCCCGTCCTCATCACG | Insert gRNA spacer sequence targeting *ptmT1* into pCRISPomyces-2 |
| *ptmT1*-up-F | AACGCTCGGTTGCCGCCGGGCGTTTTTTATCTAGATGCCCTCCAGGTGCGGGTAGTCG | Amplify the 2 kb upstream region flanking *ptmT1* |
| *ptmT1*-up-R | AAGTGGCCGGCATGACCGCCCGCCGGCCCCGC | Amplify the 2 kb upstream region flanking *ptmT1* |
| *ptmT1*-down-F | GGGGCCGGCGGGCGGTCATGCCGGCCACTTGGTCACC | Amplify the 2 kb downstream region flanking *ptmT1* |
| *ptmT1*-down-R | GCAACGCGGCCTTTTTACGGTTCCTGGCCTCTAGATGCCGAGCGACTCCGACGACACC | Amplify the 2 kb downstream region flanking *ptmT1* |
| *ptmT1*-screen-F | CGGCGAACAGTTCGGTCACCACTTC | Screen the Δ*ptmT1* mutant |
| *ptmT1*-screen-R | CTCGACAGCCTCGAAGCGGGTC | Screen the Δ*ptmT1* mutant |
| *ptmT1*-Southern-F | ATTCTCAGGTCTTCGCTCGG | Amplify the Southern probe for *ptmT1* |
| *ptmT1*-Southern-R | ACCAGATCGTCTCGTTGCTC | Amplify the Southern probe for *ptmT1* |

| **Table S2**: List of plasmids and bacterial strains used in this study | | |
| --- | --- | --- |
|  | Description | Source |
| Plasmids | | |
| pCRISPomyces-2 | CRISPR-Cas9 system for genetic editing in actinomycetes | Cobb et al., 2015 |
| pBS12129 | pCRISPomyces-2-based plasmid for disruption of *ptmR1* | This study |
| pBS12130 | pCRISPomyces-2-based plasmid for disruption of *ptmT3* | This study |
| pBS12131 | pCRISPomyces-2-based plasmid for disruption of *ptmT1* | This study |
| *E. coli* strains | | |
| NEB Turbo | Host for general cloning | New England Biolabs |
| ET12567/pUZ8002 | Methylation-deficient host for intergeneric conjugation into *Streptomyces* | MacNeil et al., 1992 |
| *Streptomyces platensis* strains | | |
| SB12029 | CB00739 Δ*ptmR1* (markerless, with a 89-nt scar) | Rudolf et al., 2015 |
| SB12051 | CB00739 Δ*ptmR1* (markerless & scarless) | This study |
| SB12052 | CB00739 Δ*ptmR1ΔptmT3* (markerless & scarless) | This study |
| SB12053 | CB00739 Δ*ptmR1ΔptmT1* (markerless & scarless) | This study |
| SB12054 | CB00739 Δ*ptmR1ΔptmT1ΔptmT3* (markerless & scarless) | This study |

| **Table S3**. Single-factor medium optimization for *S. platensis* SB12052 | | | |
| --- | --- | --- | --- |
| Carbon source | Nitrogen source | PTN (mg L^-1^) |  |
| α-Lactose, dextrin | Soybean flour | 250 ± 62 |  |
| Glycerol | Soybean flour | 187 ± 31 |  |
| α-Sucrose | Soybean flour | 12 ± 2 |  |
| α-Maltose | Soybean flour | 791 ± 119 |  |
| Soluble starch | Yeast extract | 106 ± 38 |  |
| Soluble starch | Beef extract | 30 ± 6 |  |
| Soluble starch | Cottonseed meal | 414 ± 137 |  |
| Soluble starch | Peptone | 14 ± 2 |  |
| Soluble starch | Soytone | 239 ± 18 |  |

| **Table S4**. Single-factor medium optimization for *S. platensis* SB12053 | | | | | |
| --- | --- | --- | --- | --- | --- |
| Carbon source | Nitrogen source | PTM (mg L^-1^) | PTN (mg L^-1^) | PTL (mg L^-1^) |  |
| α-Lactose, dextrin | Soybean flour | 279 ± 72 | 13 ± 1 | 17 ± 2 |  |
| Glycerol | Soybean flour | 680 ± 31 | 23 ± 3 | 32 ± 4 |  |
| α-Sucrose | Soybean flour | 643 ± 47 | 27 ± 4 | 37 ± 8 |  |
| α-Maltose | Soybean flour | 836 ± 96 | 40 ± 10 | 40 ± 5 |  |
| Soluble starch | Yeast extract | 154 ± 21 | 13 ± 1 | 5 ± 1 |  |
| Soluble starch | Beef extract | 23 ± 1 | 7 ± 1 | 2 ± 1 |  |
| Soluble starch | Cottonseed meal | 416 ± 23 | 16 ± 1 | 32 ± 1 |  |
| Soluble starch | Peptone | 14 ± 4 | 6 ± 1 | Not Detected |  |
| Soluble starch | Soytone | 413 ± 43 | 24 ± 2 | 23 ± 7 |  |

**Figure S1**. Construction and genotype verification of *S. platensis* SB12051. **a:** Schematic reflecting the removal of *ptmR1* from the chromosome of *S. platensis* CB00739 via pCRISPomyces-2-based pBS12129. **b:** Diagnostic PCR of the *ptmR1* locus using diagnostic primers shown in black in **a**. Lanes shown are (i) GeneRuler 1 kb DNA ladder (Thermo Scientific), (ii) amplification from *S. platensis* CB00739 genomic DNA, and (iii) amplification from *S. platensis* SB12051 genomic DNA. **c:** Southern blot analysis of *ptmR1* locus using probe and restriction sites shown in grey in **a**. Lanes shown are (i) DNA molecular weight marker VII, DIG-labelled (Roche), (ii) *S. platensis* CB00739 genomic DNA digested with *Bam*HI, and (iii) *S. platensis* SB12051 genomic DNA digested with *Bam*HI. **e:** Sanger sequencing verification of the Δ*ptmR1* site in *S. platensis* SB12051.


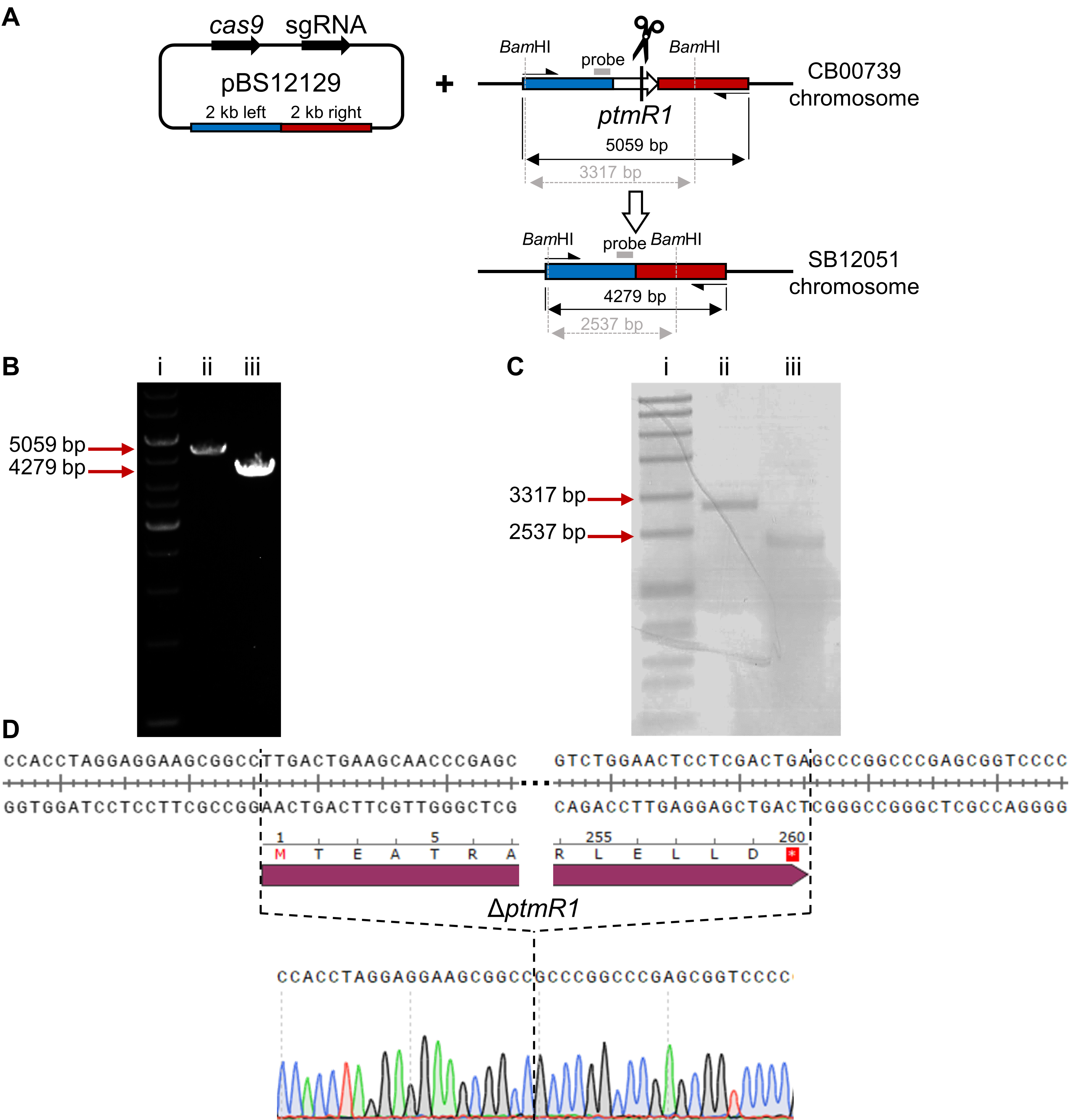


**Figure S2.** Construction and genotype verification of *S. platensis* SB12052. **a:** Schematic reflecting the removal of *ptmT3* from the chromosome of *S. platensis* SB12051 via pCRISPomyces-2-based pBS12130. **b:** Diagnostic PCR of the *ptmT3* locus using diagnostic primers shown in black in **a**. Lanes shown are (i) GeneRuler 1 kb DNA ladder (Thermo Scientific), (ii) amplification from *S. platensis* SB12051 genomic DNA, (iii) amplification from *S. platensis* SB12052 genomic DNA, and (iv) amplification from *S. platensis* SB12054 genomic DNA. **c:** Southern blot analysis of *ptmT3* locus using probe and restriction sites shown in grey in **b**. Lanes shown are (i) DNA molecular weight marker VII, DIG-labelled (Roche), (ii) *S. platensis* SB12051 genomic DNA digested with *Not*I, (iii) *S. platensis* SB12052 genomic DNA digested with *Not*I, and (iv) *S. platensis* SB12054 genomic DNA digested with *Not*I. **e:** Sanger sequencing verification of the Δ*ptmT3* site in *S. platensis* SB12052.


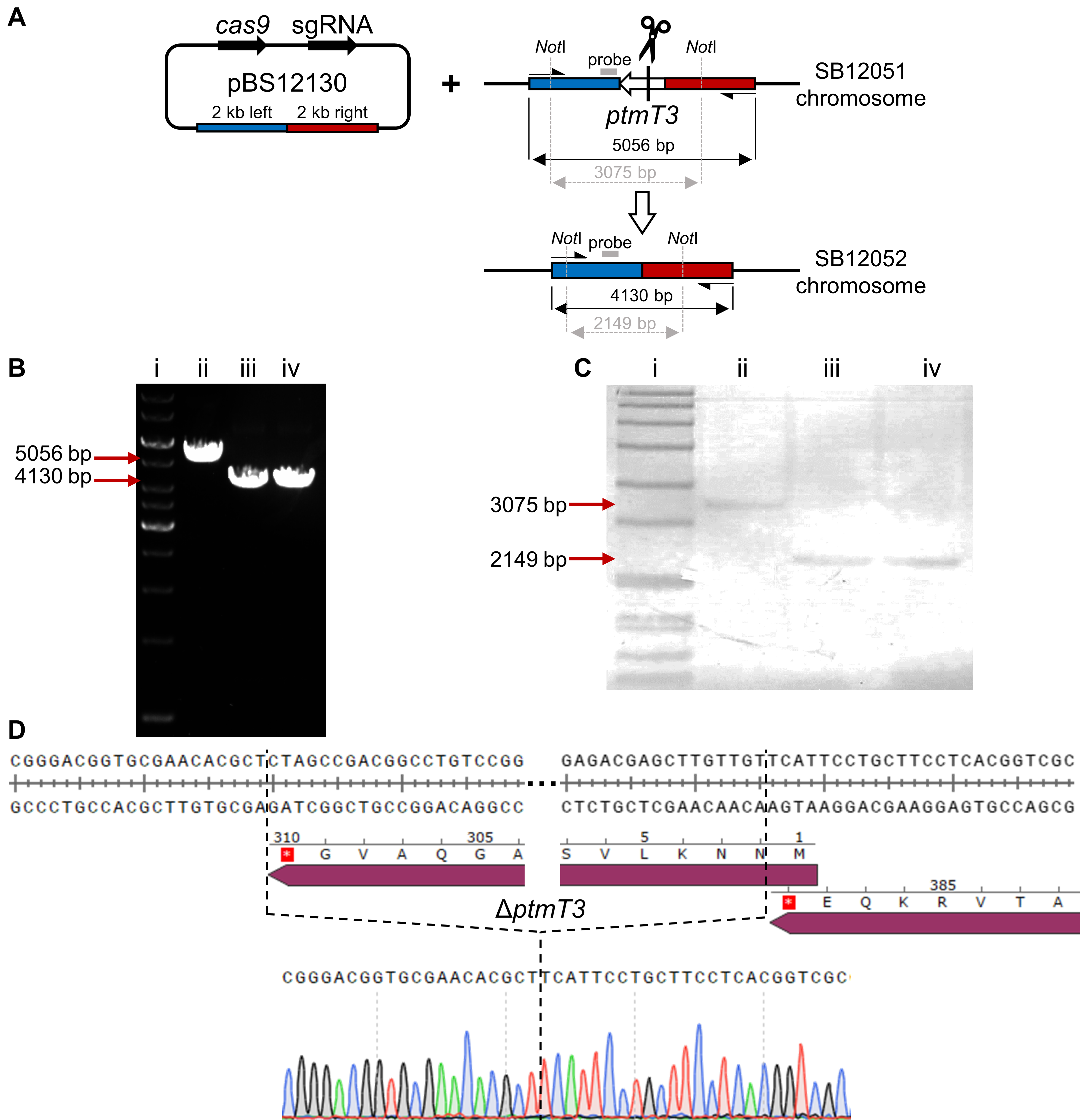


**Figure S3.** Construction and genotype verification of *S. platensis* SB12053 and *S. platensis* SB12054. **a:** Schematic reflecting the removal of *ptmT1* from the chromosome of *S. platensis* SB12051 via pCRISPomyces-2-based pBS12131. **b:** Schematic reflecting the removal of *ptmT1* from the chromosome of *S. platensis* SB12053 via pCRISPomyces-2-based pBS12131. **c:** Diagnostic PCR of the *ptmT1* locus using diagnostic primers shown in black in **a**. Lanes shown are (i) GeneRuler 1 kb DNA ladder (Thermo Scientific), (ii) amplification from SB12051 genomic DNA, (iii) amplification from *S. platensis* SB12053 genomic DNA, and (iv) amplification from *S. platensis* SB12054 genomic DNA. **d:** Southern blot analysis of *ptmT1* locus using probe and restriction sites shown in grey in **b**. Lanes shown are (i) DNA molecular weight marker VII, DIG-labelled (Roche), (ii) *S. platensis* SB12051 genomic DNA digested with *Not*I, (iii) *S. platensis* SB12053 genomic DNA digested with *Not*I, and (iv) *S. platensis* SB12054 genomic DNA digested with *Not*I. **e:** Sanger sequencing verification of the Δ*ptmT1* site in *S. platensis* SB12053. f**:** Sanger sequencing verification of the Δ*ptmT1* site in *S. platensis* SB12054.


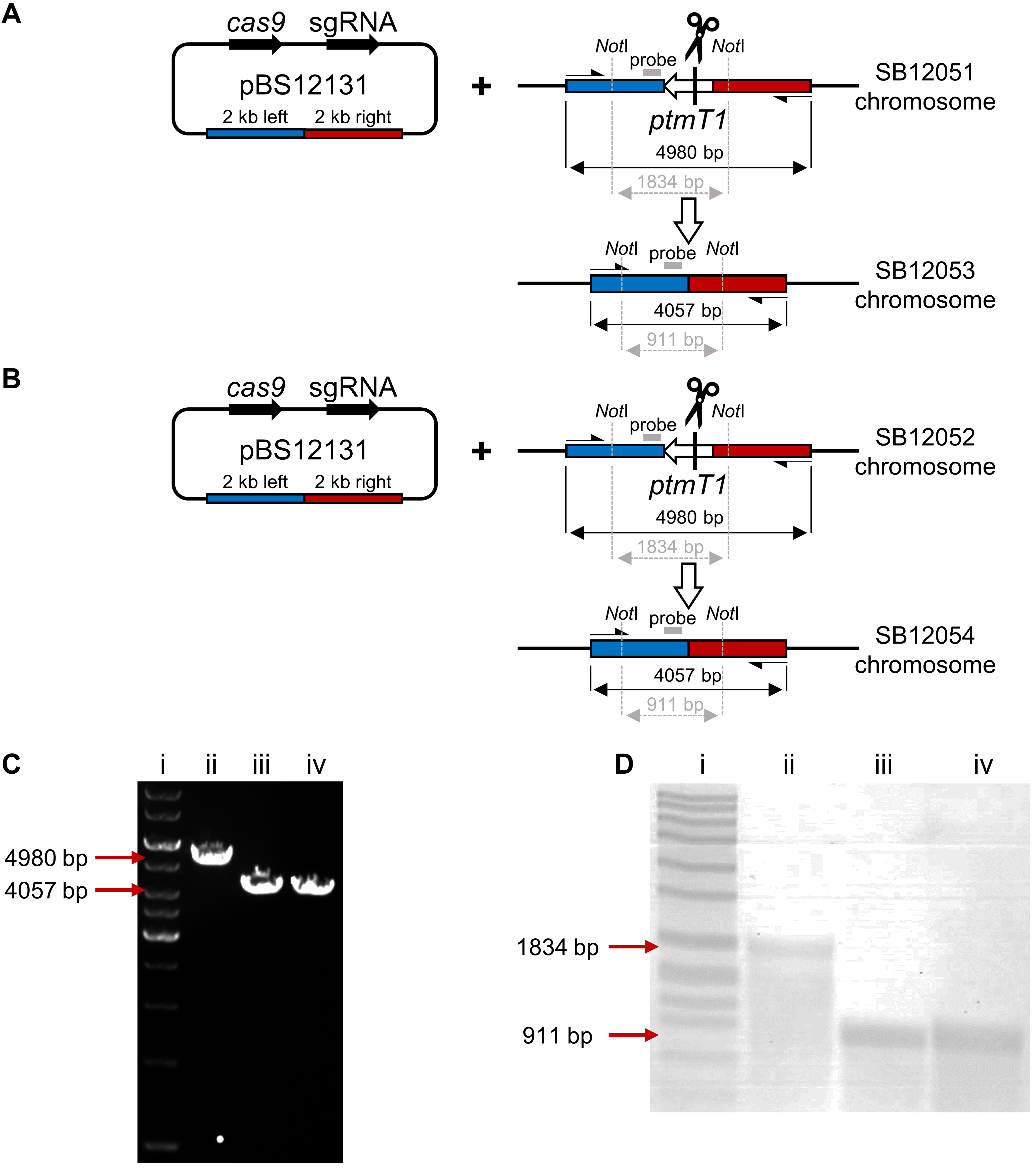


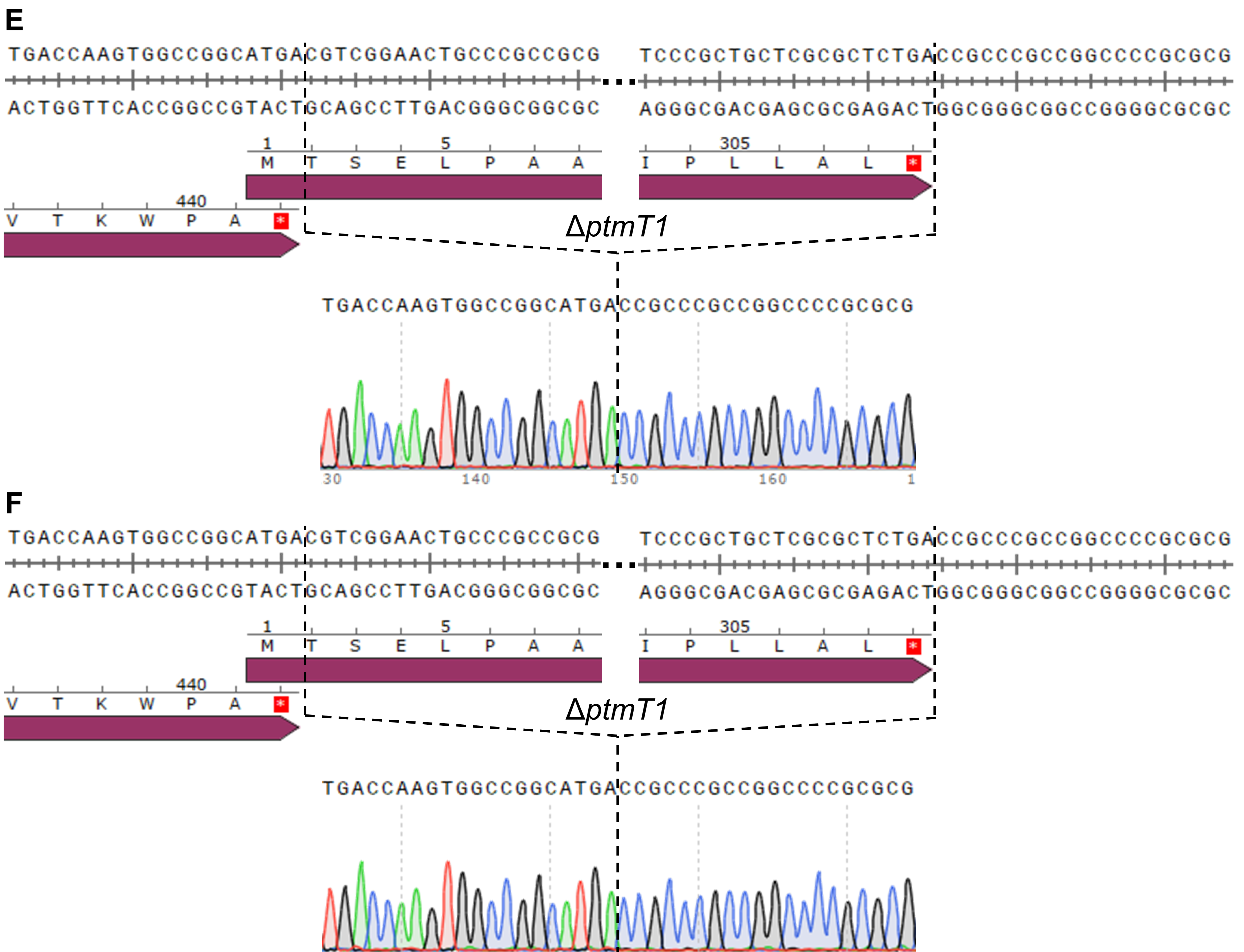


**Figure S4**. HPLC-MS profiles of engineered *S. platensis* recombinant strain fermentations. **a:** Metabolite profiles of engineered *S. platensis* recombinant strains upon HPLC analysis with UV detection at 254 nm following fermentation in PTM media. **b:** The same profiles analyzed with negative-mode electrospray ionization mass spectrometry. Shown are extracted ion chromatograms for *m/z* = 424 (black solid line, the expected mass of PTN and PTL) and *m/z* = 440 (dashed red line, the expected mass of PTM). PTM (♦), PTN (•) , and PTL (▼) standards are at a concentration of 100 μM.


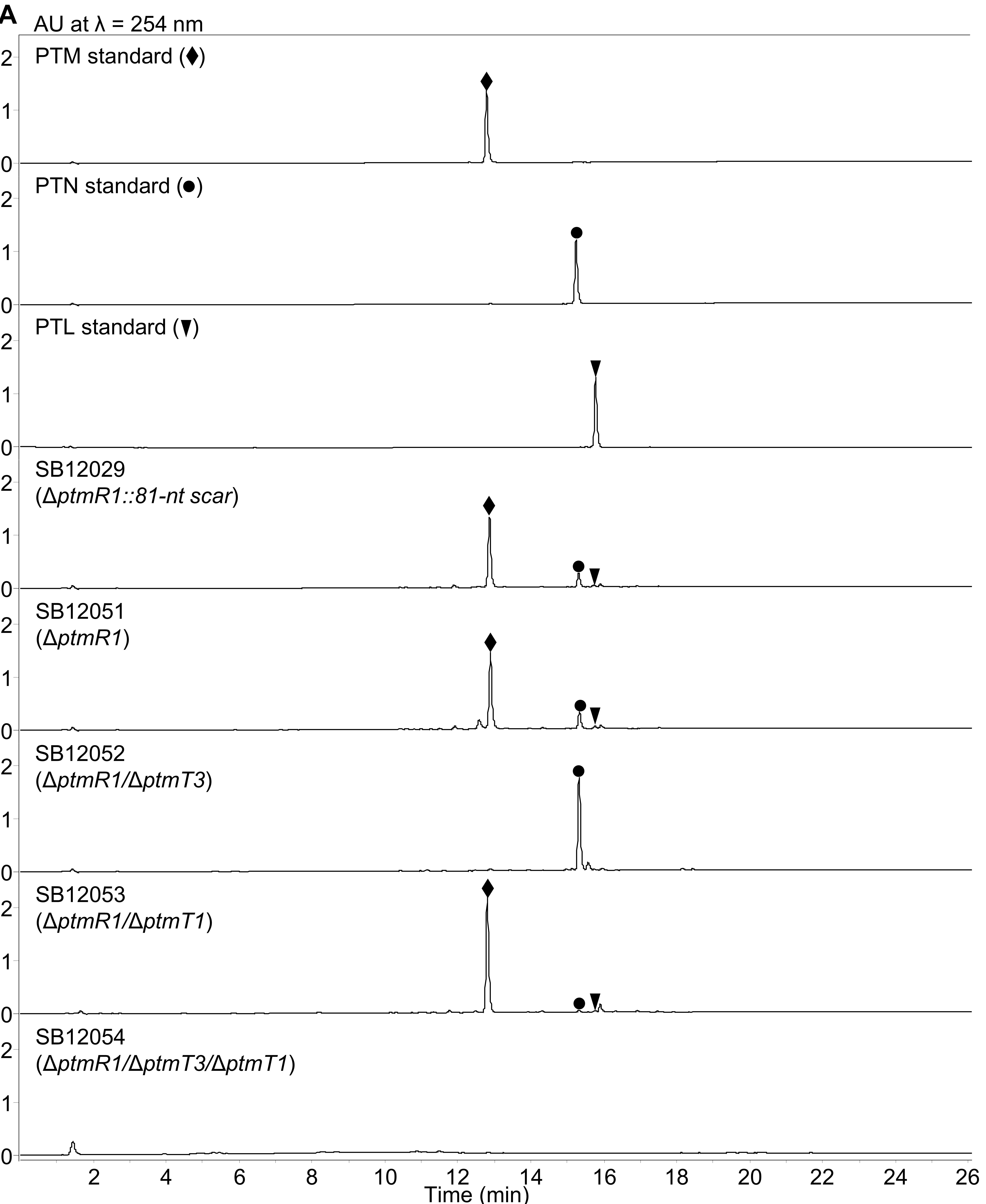


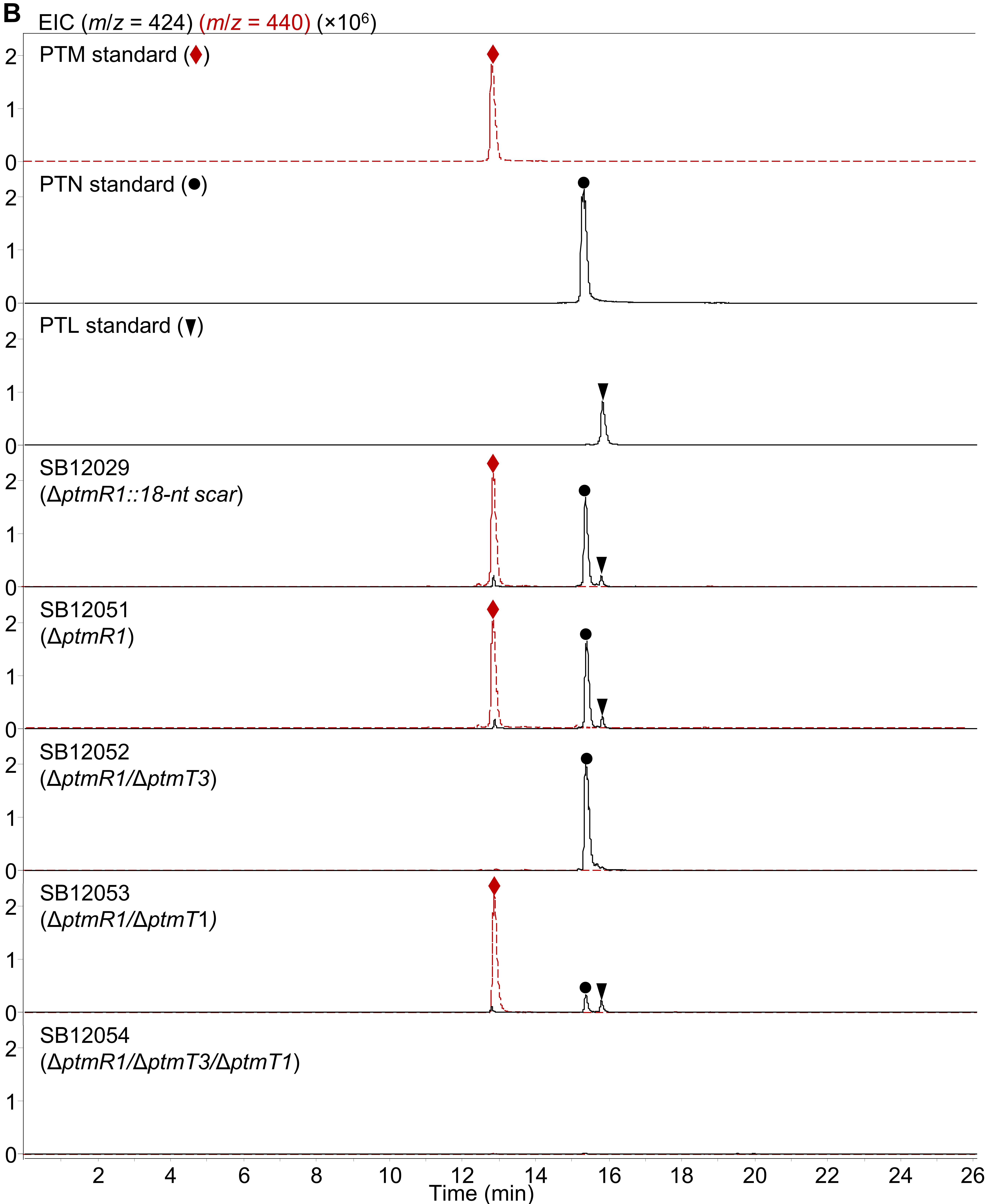


**Supplementary references**

Cobb, R. E., Wang, Y., & Zhao, H. (2015). High-efficiency multiplex genome editing of *Streptomyces* species using an engineered CRISPR/Cas system. *ACS Synthetic Biology*, *4*(5), 723–728. https://doi.org/10.1021/sb500351f

MacNeil, D. J., Gewain, K. M., Ruby, C. L., Dezeny, G., Gibbons, P. H., & MacNeil, T. (1992). Analysis of *Streptomyces avermitilis* genes required for avermectin biosynthesis utilizing a novel integration vector. *Gene*, *111*(1), 61–68. https://doi.org/10.1016/0378-1119(92)90603-M

Rudolf, J. D., Dong, L. Bin, Huang, T., & Shen, B. (2015). A genetically amenable platensimycin- and platencin-overproducer as a platform for biosynthetic explorations: A showcase of PtmO4, a long-chain acyl-CoA dehydrogenase. *Molecular BioSystems*, *11*(10), 2717–2726. https://doi.org/10.1039/c5mb00303b
